# What Is eHealth (5): A Research Agenda for eHealth Through Stakeholder Consultation and Policy Context Review

**DOI:** 10.2196/jmir.7.5.e54

**Published:** 2005-11-10

**Authors:** Ray Jones, Ray Rogers, Jean Roberts, Lynne Callaghan, Laura Lindsey, John Campbell, Margaret Thorogood, Graham Wright, Nick Gaunt, Chris Hanks, Graham R Williamson

**Affiliations:** ^5^NHS Institute for Innovation and ImprovementCoventryUK; ^4^EpidemiologyWarwick Medical SchoolUniversity of WarwickCoventry and LeicesterUK; ^3^General Practice and Primary CarePeninsula Medical SchoolExeter and PlymouthUK; ^2^Centre for Health Informatics Research and DevelopmentUniversity CollegeWinchesterUK; ^1^School of Nursing and Community StudiesUniversity of PlymouthPlymouthUK

**Keywords:** medical informatics, research priorities

## Abstract

**Background:**

In 2003, the National Health Service in England and Wales, despite its large investment in information and communication technology, had not set a national research agenda. The National Health Service has three main research and development programs: one is the Service Delivery and Organisation program, commissioned in 2003, and the others are two parallel “scoping exercises” to help set a research agenda. This paper reports on one of those projects. A parallel literature review was carried out by others and has been reported elsewhere.

**Objective:**

The objective was to explore the concerns of stakeholders and to review relevant policy in order to produce recommendations and a conceptual map of eHealth research.

**Methods:**

There were two parallel strands. For the stakeholder consultation, 37 professionals representing 12 “stakeholder” groups participated in focus groups or interviews. Discussion was prompted by eHealth “scenarios” and analyzed using thematic content analysis. Subsequently, 17 lay participants, in three focus groups, discussed and prioritized these themes. For the policy review, 26 policy makers were interviewed, and 95 policy documents were reviewed. Recommendations were subsequently reviewed in a conference workshop. Recommendations for research from both strands were combined into a conceptual map.

**Results:**

Themes from stakeholder consultation and policy review were combined as 43 recommendations under six headings. Four of these headings (using, processing, sharing, and controlling information) describe the scope of eHealth research. The other two relate to how research should be carried out (ensuring best practice is first identified and disseminated) and to the values considered important by stakeholders (in particular, measuring improvement in health).

**Conclusions:**

The scope of eHealth research (using, processing, sharing, controlling information) derived empirically from this study corresponds with “textbook” descriptions of informatics. Stakeholders would like eHealth research to include outcomes such as improved health or quality of life, but such research may be long term while changes in information technology are rapid. Longer-term research questions need to be concerned with human behavior and our use of information, rather than particular technologies. In some cases, “modelling” longer-term costs and benefits (in terms of health) may be desirable.

## Introduction

In 2002, the National Health Service (NHS) in England and Wales planned to invest over £2 billion in information and communication technology (ICT) [1]. This includes initiatives such as electronic patient records, electronic prescribing, the NHS Direct Telephone and Internet Service, and the National Electronic Library for Health [1,2]. Researchers from multiple disciplines in the UK and elsewhere had been investigating health informatics, but the NHS, despite its large investment in ICT, had not set a national research agenda for ICT.

A one-day conference in 2002 on Health Informatics Research and Development, sponsored by the research councils, Department of Health, and Department of Trade and Industry, concluded that the “lack of national strategy, capacity and career paths in health informatics have been weaknesses and remain threats to realising the informatics potential of the National Health Service…. [L]arge investment in the National Health Service and e-Science is unlikely to achieve its objectives without radical improvement in support for academic health informatics…. [This emphasizes the] importance of...clarifying the academic agenda for health informatics” [3]. The short- and medium-term challenges were seen as the following: (1) establishing the foundations of a knowledge infrastructure, (2) innovations in the clinician computer interface, (3) workable privacy protection, (4) more creation of knowledge from routinely collected data, and (5) finding the metrics of success for health informatics.

The NHS has three main national NHS research and development programs: Health Technology Assessment, New and Emerging Applications of Technology, and Service Delivery and Organisation (SDO). The SDO was launched on March 30, 2000, to consolidate and develop the evidence base on the organization, management, and delivery of health care services [4]. To respond to the needs of the “stakeholders” (service users, health professionals, and policy makers), the NHS, through its SDO research program, undertook an initial “listening exercise”[5] to produce a document outlining its overall priorities for research. It has continued to use this approach to develop and commission research [6]. It commissions a “scoping exercise” (normally a literature review and a stakeholder consultation) and then uses that in subsequent calls for proposals.

This study explored the concerns of professional and lay stakeholders regarding future developments of eHealth and reviewed relevant policy to produce recommendations for eHealth research. A parallel literature review was carried out by others and has been reported elsewhere [7-9].

## Methods

### Study Design

**Figure 1:**
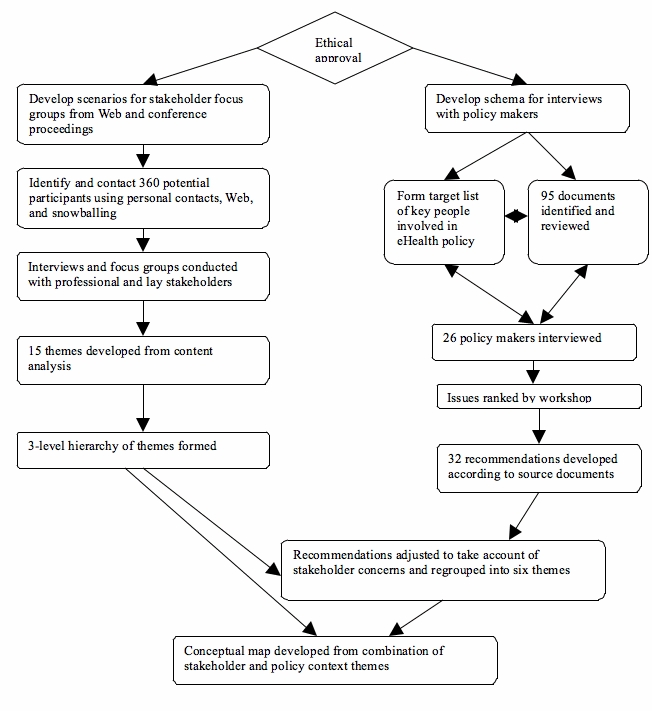
Parallel methods of stakeholder consultation and policy review

The study was reviewed and approved by the SouthWest Multi-Centre Research Ethics Committee and the University of Plymouth Faculty of Health and Social Work Ethics Committee. Data collection was carried out between November 2003 and June 2004. There were two parallel strands (Figure 1):

1. Stakeholder consultation: Focus groups and interviews with “professional” stakeholders generated themes that were subsequently prioritized by lay participants.
2. Policy context review: Policy makers were interviewed and policy documents were reviewed in order to produce recommendations that were subsequently reviewed in a conference workshop.

The themes and research questions arising from the stakeholder consultation and policy context review were compared, and recommendations from policy context were adapted to take account of stakeholder concerns. Diagrams were developed to bring together stakeholder and policy maker views of the scope of eHealth research.

### Stakeholder Consultation

In all, 12 groups (30 in each group) of professional stakeholders were contacted via email. Potential participants were identified by Web searches and by “snowballing” from existing contacts, trying to get geographical coverage within England. Stakeholders were defined as the following:

- NHS eHealth innovators and implementers
- University researchers in health informatics
- NHS staff in primary care
- NHS staff in secondary care
- NHS primary care trust managerial staff
- NHS acute trust managerial staff
- Suppliers of ICT to the NHS
- Professional organizations and royal colleges
- Informatics trainers
- Governance and other regulators
- Charities and other information providers
- Other NHS managers

These 360 people were sent an email inviting them to take part in the study, with a consent form to return to the researcher via email or post if they agreed to take part in the study. We asked professional participants to rank themselves on a four-point scale (from “I am pretty sceptical that eHealth will have any benefits at all” through to “I am very positive that eHealth can help improve the NHS if used appropriately”) as a rough guide to attitudes toward eHealth (Table 1).

**Table 1:** Professional participants: locations and eHealth attitudes

**Stakeholder Group (number of participants)**	**Location in England**	Self-Rating on eHealth*
Innovators and implementers (5)	South West	3
	South East	4
	South East	3
	South East	4
	South West	3
Academic researchers in eHealth (4)	Midlands	3
	Midlands	3
	South West	3
	South East	3
NHS staff in primary care (3)	South West	3
	South West	4
	South West	4
NHS staff in secondary care (4)	South East	3
	South East	3
	South West	4
	South East	3
Primary care managerial staff (1)	South West	3
Acute care managerial staff (1)	South East	3
Suppliers (3)	South East	3
	South West	4
	North	3
Royal colleges (3)	South East	3
	South East	4
	South East	4
Informatics trainers (5)	South East	3
	Midlands	3
	Midlands	3
	Midlands	3
	South East	3
Governance (3)	South East	3
	South East	4
	South East	3
Charities and other providers (2)	Midlands	3
	South East	3
Other NHS managers (3)	South East	3
	South West	4
	North	3

^*^ Self-ratings: (1) I am pretty sceptical that eHealth will have any benefits at all. (2) I think that there could be some possible benefits to eHealth methods but on balance think that it is unlikely that the benefits will outweigh the costs. (3) I think that there are definitely benefits to eHealth but that we need to choose and develop methods carefully. (4) I am very positive that eHealth can help improve the NHS if used appropriately.

A convenience sample of lay participants was recruited via snowballing from contacts in a local children’s nursery and from older friends of the research team. Potential participants were sent study information sheets and consent forms either via email or post. Two groups of older people and one group of parents took part in the study.

Scenarios depicting the current or future use of eHealth technologies were constructed to prompt discussion among the professional groups of the relevant themes regarding the use of eHealth technologies. Subject matter of the scenarios was developed from the content of news reports, informatics conference proceedings, and general Web searches. Both patient- and professional-centered scenarios were developed in order to achieve a balance of perspectives. The research team developed and discussed 32 scenarios: 15 were omitted, 7 were added, and a number were reworded to ensure neutrality in presentation.

In total, 24 scenarios (see Multimedia Appendix 1) were allocated to the 12 professional groups using a balanced incomplete block design [10]. Each group had four scenarios, and each scenario was used twice. A semistructured schedule based on the scenarios was constructed for use either as focus group or interview prompts. Some scenarios described patients being monitored by an implanted device that sent physiological information to hospital, a family doctor booking a hospital appointment during consultation in primary care, and a woman having an antenatal ultrasound in the community with expert diagnosis from abroad. Textbox 1 provides four examples of scenarios used as prompts in telephone focus groups. The topics covered in the scenarios included patients’ use of the Internet to order prescriptions, arrange doctor’s appointments, or join patient discussion forums. Other topics were about patients accessing their own health record, assessing the quality of a website, using a digital interactive television for a program on multiple sclerosis, or using a public access touch screen health information point.

**Four examples of scenarios used as prompts in telephone focus groups**

1. Ordering prescriptions: Sam, 45, drops off a repeat prescription for his high cholesterol medication every month and has a check-up routinely every three months. Recently, the local pharmacy and Sam’s family doctor have started a scheme whereby patients can order their repeat prescriptions online, thereby relieving the burden on administrative staff at the surgery (primary care health center). Following an order being made by a patient, the pharmacy provides the doctor with a list of repeat prescriptions, which the doctor approves or not. The pharmacy then sends an email to Sam when his medication is ready for collection.
2. Use of implanted device: James, a diabetic, has an implanted device that measures his blood glucose level and transmits this reading to the hospital. If the reading is below a certain level, James is contacted on his mobile phone by an automated system. Recently, the hospital received a signal that James’s blood glucose had dropped to 1.5. The doctor was alerted and visited the patient to review his medication.
3. e-booking: Peter, aged 45, attends an appointment with his doctor about his recent weight loss. His doctor decides that Peter should be referred for an appointment at the hospital and uses the new e-booking system. Upon inputting Peter’s details into the system, an appointment was set up immediately, and Peter was able to leave the surgery with his hospital appointment arranged.
4. Wireless technology: Ann Young is a district nurse who uses a palmtop with wireless access to the internet and PCT intranet. Ann regularly uses her palmtop in order to ask advice of her colleagues or to obtain test results, and she now views her palmtop as an invaluable resource. At the next practice meeting, Ann intends to present the benefits of using a palmtop to her colleagues.

### Policy Context

A parallel, two-stage process was used to review the policy context. First, policy makers were interviewed and policy documents were identified and reviewed. The contents of documents and interview notes were categorized under English policies on ICT specific to health, English health policies influencing eHealth, nonhealth policies influencing eHealth, and European Union policies influencing eHealth. The reviewers focused on seven specific topics: (1) birth to death records, (2) country-wide access to quality health advice, (3) application of ICT to pharmacy, (4) telemedicine, (5) reduction of adverse incidents, (6) confidentiality, and (7) health data cards. In a second stage, 28 recommendations from the policy makers and documents were reviewed in a health informatics conference workshop by 60 participants using discussion and an interactive voting system. Participants scored each recommendation for relevance to the needs of the NHS (using a nine-point scale from “not relevant” to “highly relevant”). Recommendations with “middle scores” were discussed with the audience in more detail to obtain their views and decide if the recommendation needed to be worded more clearly.

### Synthesis and Conceptual Mapping

Two members of the research team (RJ and LC) independently ranked the correspondence between the stakeholder concerns and the policy context recommendations on a scale of 1 (no correspondence) to 3 (strong correspondence). Analysis showed there to be strong agreement between the researchers. Stakeholder concerns, particularly of “technology meeting needs and improving health and quality of life,” were not consistently addressed by the policy context recommendations. Recommendations were adapted to take stakeholder concerns into account and were regrouped from “source-oriented” to “research-oriented” groupings. The new policy context recommendations were agreed upon between team members by telephone conference discussion. The two lists, one from stakeholder consultation and the other from the policy context review, were then reviewed again and similar areas of research were grouped. Reference was also made to the work of the Scottish Consumer Health Informatics Network [11]. We concluded that the scope of eHealth research could be described by a simple block diagram with four elements with linked areas of “best practice.” The recommendations were regrouped according to this “conceptual map.”

## Results

### Subjects and Sources

In total, 37 (10%) professional stakeholders consented to take part in the study and were consulted either via telephone focus group (25), telephone interview (6), videoconference (4), or in-person interview (2). There were 17 lay people (12 older people and 5 parents of young children) that took part in in-person focus groups. We interviewed 26 policy makers and identified and reviewed 95 policy documents.

### Validity of Methods

One of the limitations of stakeholder consultation is that participants have to be sufficiently interested in the topic to take part. None of the 37 professional participants were “sceptical that eHealth will have any benefits at all,” and none thought “there could be some possible benefits to eHealth methods but on balance think that it is unlikely that the benefits will outweigh the costs.” On the other hand, 27 thought “that there are definitely benefits to eHealth but that we need to choose and develop methods carefully,” and 10 were “very positive that eHealth can help improve the NHS if used appropriately.”

### Scope of eHealth

**Figure 2:**
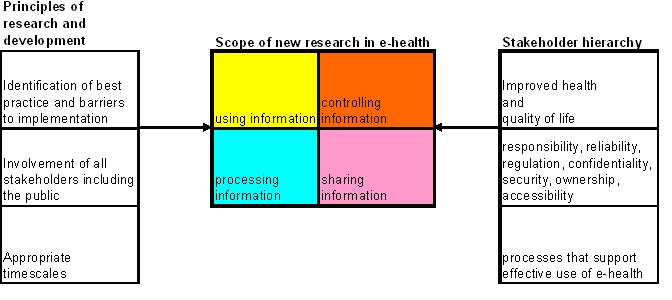
Scope of eHealth research

The research questions identified by stakeholders and policy review fell into six groups. Four of these (using, controlling, processing, sharing information) were used to describe the “scope” of eHealth. The other two groups of research questions fall under principles of research and development and stakeholder hierarchy (Figure 2).

### Principles of Research and Development

Both stakeholders and policy makers referred to many examples where, before innovative approaches are introduced, best practice procedures and barriers to implementation should be identified, and where professional and public stakeholders should be involved in research and development. A number of areas were suggested (Textbox 2).

**Seven research aims related to the identification and implementation of best practice in eHealth**

1. Informatics training for health professionals, identification and exploration of examples of best practice to see how these can be disseminated to achieve improved health care, exploration of the attitudes of health professionals toward such training and use of the skills acquired in practice
2. Working practices in other sectors (eg, e-business) to identify best practice and barriers to similar uses of ICT in the health sector
3. Web-based services for citizens in other sectors to see what lessons can be learnt on when to implement eHealth solutions for patients
4. Research on telemedicine (eg, using coronary heart disease or cancer services) and barriers to its implementation
5. The costs and benefits (including improved patient safety) of hospital systems that combine e-prescribing, order entry, decision support, bar coding for medication management, and robotic dispensing
6. NHS procedures that aim to safeguard confidentiality of patient data and disseminate best practice
7. Assessment of the experience of UK citizens accessing health care in other countries (and vice versa) and identification of where health and other outcomes could be improved through the use of ICT

### Scope of New Research in eHealth

#### Using Information

Information is used in decision support, in the organization of services, for reassurance of professionals and patients, and in information-based therapies. Four research aims (Textbox 3) from the policy context review concerned the use of information in decision support.

**Four research aims related to the way information is used**

1. To assess clinicians’ and patients’ perceptions of the benefits and barriers to using decision support tools—in particular, to compare clinicians who use decision support tools with clinicians who do not
2. To assess the quality of information available from repositories of health data and to assess how it can be legally, ethically, and cost-effectively aggregated for public health policy and decision support
3. To explore the costs and potential benefits of birth to death records in relation to decision making and other aspects of health care and to identify policy changes required to achieve them
4. To review decision support and expert systems used in the NHS to ascertain their impact on patient services

#### Sharing Information

Both stakeholders and the policy context review identified a large number of research questions related to sharing information (Textbox 4). These included how information should be shared across sites (eg, between hospital and home), across sectors (eg, between social services and NHS), and between different professional (and patient) groups (eg, between doctors, nurses, dentists, patients).

**Thirteen research aims related to the way information is shared**

1. To examine how the NHS can work with other information and education providers to facilitate patient involvement in eHealth
2. To explore patient attitudes toward initiatives of patient involvement in eHealth
3. To identify the extent to which implanted or wearable technology removes patients’ control of their condition and to identify how ICT may best be used to encourage and facilitate patients to take responsibility for their health
4. To investigate the extent to which recently introduced information technology–based systems (such as e-booking) increase patient expectations and consequently decrease satisfaction if those expectations are not met
5. To investigate the efficacy of developing a code of collaboration under which organizations can explicitly share data and input to health records consistently, unambiguously, and sensitively
6. To determine how we can best deal with combining multiple sources of data, dealing with apparently conflicting information from different sources, with minimum patient risk, minimum cost, and patient consent and confidence
7. To examine the costs and benefits of cross-sectoral records and patient safety issues associated with cross-sectoral working
8. To investigate how ICT can best contribute to pharmacy clinic services sharing data between the NHS and patient
9. To investigate the potential of eHealth to enable effective interfaces, for example, between health and social care, local specialists and specialist services, care givers and professionals
10. To investigate the costs and benefits of using different technologies to support community-based staff (eg, notepad computers, electronic links to supporting organizations, teleconferencing in cancer services)
11. To explore the changes in work patterns, the potential for patient involvement, and legal issues in home care (eg, for older people)
12. To investigate ICT use in multisite work in relation to such issues as culture change, governance, health professional training, patient expectations, and changes to health outcomes
13. To determine the costs and benefits of the use of health data cards

#### Controlling Information

This group of concerns was ranked second most important by stakeholders. Ten research aims (Textbox 5) incorporated issues of control, accessibility, reliability, confidentiality, security, ownership, and regulation.

**Ten research aims related to the way information is controlled**

1. To investigate how health professionals and patients discriminate between reliable and unreliable information
2. To examine the circumstances in which regulation of information provision and use is necessary and, further, when education and empowerment of professionals is a more effective option—additionally, what are health professional and patient attitudes toward the regulation of health information?
3. To investigate the extent to which health professionals advise patients of reliable sources of information on the Web, television, and other media, and further, to examine the level of preparation and support that health professionals require to provide such advice and to examine patients’ expectations of this advice
4. To determine the site of responsibility if health care errors are made as a result of information transfer
5. To explore how social organization and different technologies can be used to help prevent inequity of access to information for both patients and professionals, and to identify initiatives whereby groups traditionally considered to have restricted access have successfully achieved training and access to new technologies
6. To explore health professional and patient attitudes toward ownership and sharing of data
7. To develop and test guidance on regulation and responsibility
8. To examine the costs and benefits of different ways of addressing equity to inform citizens
9. To investigate ways (quality marks, portals, patient and health professional training) to assist the public in obtaining quality information from the Web
10. To investigate patients’ knowledge and views on confidentiality and their attitudes as to how their data should be used (eg, in research) in terms of potential benefits to health and quality of life

#### Processing Information

This covers a range of issues, including how best to present information (eg, should it be tailored for different users) and where it might be presented (eg, should it be sent to the user [push], or should it wait until the user seeks it [pull]). It also includes the coordinated integration of information derived from a variety of sources, as demonstrated in the electronic ordering and home delivery of medicines (Textbox 6).

**Seven research aims related to the way information is processed**

1. To identify what extent health information should be tailored to the needs of certain groups of patients, professionals, or individuals
2. To examine the costs and benefits of providing information in different locations (eg, mobile versus static for professionals, NHS versus home for patients)
3. To investigate how information can be better integrated so that patients can, for example, access their own medical record on the Web, obtain relevant and validated information about it, and order a prescription
4. To identify instances or circumstances when patients want to enquire about health information through known professionals (eg, family doctor) and when they want to use an anonymous source
5. To investigate what services patients desire for electronic ordering and home delivery of medicine and how they can be delivered safely, equitably, and cost-effectively
6. To identify how eHealth technologies can enable or improve family support for seriously ill children and provide just-in-time information tailored to individuals
7. To examine the costs, benefits, attitudes toward, and the use of, ICT support in their homes for patients with severe chronic disease (eg, video links to NHS and voluntary services, smart cards with patient records)

### Hierarchy of Stakeholder Concerns

The overriding concern of stakeholders was that spending money on eHealth should be worthwhile and should lead to improved health and quality of life. Particular research aims suggested by the data included “to review the costs and benefits of a range of recent eHealth applications, including the modelling of new forms of care made possible by ICT support,” and “to present those examples of eHealth applications, shown to have a demonstrable effect on improved health and quality of life, to professional and public stakeholders to obtain their views as to the nature of the most appropriate investment in eHealth.” In addition, the stakeholders identified themes concerned with controlling information (responsibility; reliability; regulation; accessibility; confidentiality, security, and ownership) as being particularly important, and so placed them on the second level of a hierarchy of concerns.

## Discussion

In the context of the British health service, which is mostly free at the point of delivery, the overriding concern of stakeholders was that spending money on eHealth should be worthwhile and should lead to improved health and quality of life. At first this appears an unremarkable finding. However, although such an aim is part of the political rhetoric and may be an unstated assumption of policy documents in the United Kingdom, it is not often explicitly addressed in service development and use of information and communication technology. It is significant, for example, that the NHS recently funded a program originally called the National Programme for Information Technology, rather than a program for eHealth. (Subsequently, the program has been renamed Connecting for Health).

On the other hand, we know that doing research that can show a difference in health as a result of an eHealth intervention is difficult, partly because partial implementation of an e-booking system or a hospital information system is difficult. Gold standard randomized trials whose results can be generalized for widespread implementation are very difficult. In addition, to see changes in health or to measure cost benefit is slow and expensive, made more so now in the United Kingdom by the time needed for increased levels of ethical and research control and approval [12,13]. Thirty years ago, Blum noted that half the papers about computer applications concerned systems that were no longer operational [14]. We all know that ICT changes become ever more rapid. Research, therefore, has to be more about human behavior and how we use information and less about specific organizational or technological environments. It is essential to recognize the difficulties of addressing stakeholders’ needs by measuring change in health outcomes.

In some cases, modelling the longer-term costs and benefits (in terms of health) may be desirable. As systems continue to evolve, the health benefits may be seen not in the immediate change, but in a future evolution made possible by the initial change [15-17].

Many of the recommendations derived from both the stakeholder consultation and policy review confirmed the need to identify best practice and the barriers to implementation of that best practice. There are many examples of medical informatics research with demonstrable benefit which decades later still wait to be implemented more widely. For example, computers have been used successfully for patient interviews for nearly 30 years. Slack et al first reported on a computer-based medical history system in the *New England Journal of Medicine* in 1966 [18]. Yet, despite numerous research reports [19], the method has not been routinely adopted.

Rogers’ description of the diffusion of innovations [20] is well known, but stakeholders and policy makers want to see this process accelerated. The recommendations included, for example, identifying and exploring examples of effective informatics training for health professionals to see how these can be disseminated, or investigating working practices in other sectors (eg, e-business) to identify best practice and barriers to similar uses of ICT in the health sector.

In discussion, professional stakeholders often reverted to their role as patient or consumer rather than, for example, speaking as a supplier of ICT or from the point of view of primary care. Thus, although we sampled from all segments of the stakeholder population, there were no obvious differences between the different types of stakeholder. (Our sample was small, however, making our power to detect differences limited.)

The four categories which emerged as the grouped themes from the data are similar to classifications and descriptions found in textbooks of health informatics (eg, [21]). Blum, in a personal review of Medical Informatics in the United States, 1950-1975, presents a historical table of the Scope of Medical Computing from 1950 to 1980 using the three headings Data, Information, and Knowledge Applications [14]. He said, “Within a category, research begins only after the supporting technology is mature enough to support it beyond the conceptual level.” His table showed that he viewed data applications in the 1980s as refined, information applications as mature, and knowledge applications as prototype.

Four limitations of our study are the following: (1) We were not able to recruit people who were very sceptical about the potential of eHealth. However, we think that a more sceptical sample would be likely to have expressed similar concerns about improved health and value for money. (2) We did not have equal representation for the 12 predefined groups. However, we achieved coverage (although, in some cases, only one member; see Table 1) for all groups. Furthermore, as most participants often “reverted” to their role as patient in the discussion, our original idea that different professional groups might have certain biases and try to put forward ideas to their advantage seemed wrong (although numbers were small to detect any differences between groups). (3) Our lay sample was recruited only in the South East and the South West of England and did not include any people from ethnic minorities. We have no knowledge or hypotheses about how views may differ in groups not represented. (4) An eHealth agenda derived from policy review and stakeholder consultation in a country with a state-run health service may not transfer well to countries with private or insurance-based systems.

The purpose of our study was to produce a conceptual map and research agenda for eHealth based on stakeholder views and policy review. In a parallel SDO project, Pagliari et al [7-9] developed recommendations based on a review of existing academic and wider evidence sources, indicating the scope of the eHealth concept, the effectiveness of eHealth innovations, issues for implementation, and future directions for eHealth. Their research evidence is grouped by four broad technological categories:

1. Decision support tools for patients and clinicians
2. Networked digital technologies (Internet) used by patients (eg, for information, self-management, or peer support) and professionals (eg, for interprofessional communication, education, or communication with patients)
3. Computerized patient records, including issues relating to patient access and confidentiality and influences on health care delivery
4. Telemedicine and telecare

Their results are also interpreted with respect to the following broad content areas:

1. Specific research needs (evidence of effectiveness in specific areas)
2. Generic research needs (eg, methodological challenges to eHealth research, factors affecting implementation, effects on behavior and relationships, educational interventions, health inequalities, alternative delivery media, risks to the health service and society, role in self-care, and consumer empowerment)
3. Challenges for implementation (demonstrating impact, high-level support, strong project management, stakeholder engagement, the digital divide, ensuring credibility and quality, ethical, security and privacy issues, standards)
4. Emerging trends and future directions (eg. personalized and tailored systems, new technological advances for the information management and care facilitation, and delivery modes)

While differences in emphasis were expected due to the methodology of each study, the clear parallels between the results offer support for our recommendations. Our conceptual map, which has come from stakeholder discussion and policy review, also helps to put both our own and other detailed recommendations into a framework concerned with information and how we use it.

## Multimedia Appendix 1

List of 24 scenarios.

## Abbreviations

ICT: information and communication technology
NHS: National Health Service
SDO: Service Delivery and Organisation

## References

[1] Department of Health (2002). Delivering 21st Century IT Support for the NHS: National Strategic Programme.

[2] Department of Health (2000). The NHS Plan. A Plan for Investment. A Plan for Reform.

[3] Medical Research Council Proceedings of the Workshop on Health Informatics Research and Development; July 18, 2002. London, UK: Royal College of Obstetricians and Gynaecologists.

[4] NHS Service Delivery and Organisation (SDO) Programme Home page.

[5] Fulop N, Allen P (2000). Service Delivery and Organisation National Listening Exercise: Report of the Findings.

[6] Lomas Jonathan, Fulop Naomi, Gagnon Diane, Allen Pauline (2003). On being a good listener: setting priorities for applied health services research. Milbank Q.

[7] Pagliari C EH1 E-Health Scoping Exercise. Review of the Traditional Research Literature.

[8] Pagliari C EH1 E-Health Scoping Exercise. Review of the Non-Traditional Research Literature.

[9] Pagliari Claudia, Sloan David, Gregor Peter, Sullivan Frank, Detmer Don, Kahan James P, Oortwijn Wija, Macgillivray Steve (2005). What is eHealth (4): a scoping exercise to map the field. J Med Internet Res.

[10] Armitage P, Berry G (1987). Statistical Methods in Medical Research.

[11] Marsden J, Jones R Establishing and maintaining the Consumer Health Informatics Network for Scotland (CHINS). Health Care Computing 2004:169-177.

[12] Elwyn Glyn, Seagrove Anne, Thorne Kym, Cheung Wai Yee (2005). Ethics and research governance in a multicentre study: add 150 days to your study protocol. BMJ.

[13] Hearnshaw Hilary (2004). Comparison of requirements of research ethics committees in 11 European countries for a non-invasive interventional study. BMJ.

[14] Blum BI (1990). Medical Informatics in the United States, 1950-1975. In: Blum BI, Duncan K, editors. A History of Medical Informatics. Proceedings of the ACM Conference on the History of Medical Informatics; Bethesda, Maryland; November 5-6 1987.

[15] Friedman CP, Wyatt JC (1997). Evaluation Methods in Medical Informatics.

[16] Mumford E (2000). A socio-technical approach to systems design. Requir Eng.

[17] Jones RB, Hedley AJ (1986). Evaluation of a diabetes register and information system. In: Richards B, editor. Current Perspectives in Health Computing.

[18] Slack W V, Hicks G P, Reed C E, Van Cura L J (1966). A computer-based medical-history system. N Engl J Med.

[19] Jones R, Knill-Jones RP (1994). Electronic patient record project: direct patient input to the record. Report for the Strategy Division of the NHS Information Management Group.

[20] Rogers E (1995). Diffusion of Innovation. 4th ed.

[21] World Health Organization (1988). Informatics and Telematics in Health. Present and Potential Uses.

