# Supplementary material for: What Is eHealth (5): A Research Agenda for eHealth Through Stakeholder Consultation and Policy Context Review
Source: J Med Internet Res. 2005 Nov 10;7(5):e54. doi: 10.2196/jmir.7.5.e54 (PMC1550684; doi:10.2196/jmir.7.5.e54)
Supplement: Supplementary file 1 [file jmir_v7i5e54_app1.pdf]

Final List of 24 Scenarios

1. Ordering prescriptions:
Sam, 45, drops off a repeat prescription for his medication for high cholesterol every month and has a check up routinely every three months. Recently the local pharmacy and Sam's surgery have started a scheme whereby patients can order their repeat prescriptions online, thereby relieving the burden on the administrative staff at the surgery. Following an order being made by a patient, the pharmacy provides general practitioners (GPs) with a list of repeat prescriptions to which they indicate their approval or otherwise for its administration. The pharmacy then sends an email to Sam when his medication is ready for collection.

2. Use of implanted device:
James, a diabetic, has an implanted device that measures his blood glucose level and transmits this reading to the hospital. If the reading is below a certain level, James is contacted on his mobile phone by an automated system. Recently, the hospital received a signal that James's blood glucose had dropped to 1.5. The GP was alerted and visited the patient to review his medication.

3. Test card for blood tests:
Christine, a practice nurse, has recently involved a number of her patients in a trial of a new test card, which enables diabetic patients to perform blood tests in their own home. The test card, developed by SmartSensor Telemed Ltd, contains a sensor consisting of an enzyme that "eats" glucose and creates a signal. This signal is then sent to the hospital where the results of the blood test can be straightforwardly added to the patients' electronic records. Due to the success of the trial from both the patients' and the professional's point of view, the practice managers are keen to establish the use of this system for all of the diabetic patients in their care. All diabetic patients, regardless of whether they participated in the trial, will be sent a letter inviting them to make use of this new scheme for a nominal fee of £10.00.

4. Arranging appointments:
Anna, 69, has received a letter from her GP's surgery detailing a new method of making appointments over the Internet with GPs, practices nurses, and health visitors at her surgery. The letter provided information about the surgery's new website and how to use the new booking system. Although Anna has some experience using the Internet from helping her grandson with his homework, she does not have direct access to a computer. Further, although from reading the letter, Anna is aware that she could access this system from any computer with an Internet connection using a username and password, she is worried about using computers in the library or in an Internet cafe.

5. Discussion forums:
Man-Li, a recently diagnosed insulin-dependent diabetic, has recently learned of an Internet-based discussion forum for individuals with her condition. After a week of regular visits to the forum, Man-Li feels less isolated and believes that she is better informed of her condition and the treatment that she is receiving.

6. Use of digital interactive television:
Sarah, 22, is planning to watch an interactive television program on multiple sclerosis as her mother has recently been diagnosed with the condition. Sarah's television system allows her to not only to watch the program, but also to send in messages and questions while the show is being broadcast. During the program, the issue of caring for relatives is raised by a member of the panel. As this is an issue that Sarah has been thinking about recently, she sets about sending a question to the panel.

7. Dissemination of health information:
Lisa is 29 years old and pregnant with her first baby. As she is in the final trimester of pregnancy, Lisa needs to let her employer know how long she will be taking for maternity leave after her baby is born. As breast-feeding her baby is very important to Lisa, she decides to search the Web for up-to-date information regarding the optimum period of time to breast-feed. As she would prefer to exclusively breast-feed, this would affect the length of time required for maternity leave. The search produced conflicting results with one government-affiliated website suggesting an optimum period of four months and an international charity-based website recommending exclusive breast-feeding for six months. Lisa noticed that the first website had been updated three months previously, whereas the second website had been updated only the week before. However, as it was unclear which information on the website had been updated, Lisa remained confused as to the correct information to rely upon.

8. Health information point for students:
A university health centre has received funding for a touch screen electronic health information point to be installed in the Students' Union. The information point would contain information on sexual health and drug and alcohol abuse, as well as general public health information and details of university-based and local health services.

9. Use of Internet to gather health information:
Kira, 3, has been sent home from her day nursery as she has been discovered to be carrying live head lice. The nursery is unable to admit any child who is either carrying live lice or eggs. Kira's mother needs to treat her daughter's condition quickly in order to both alleviate the problem and to return to work. Although advice was provided at the nursery about products to buy from the pharmacy, Kira's mother feels that she needs to be better informed of her options and the products available before she makes a choice. She therefore decides to use the Internet to gather information but is unsure where to start.

10. e-Booking:
Peter, aged 45, attends an appointment with his GP about his recent weight loss. The GP decides that Peter should be referred for an appointment at the hospital and uses the new e-booking system. Upon inputting Peter's details into the system, an appointment was set up immediately, and Peter was able to leave the surgery with his hospital appointment arranged.

11. Teleconsultations/remote monitoring devices/diagnosis:
Jayne is 40 years old, single, and 18 weeks pregnant. Although this is her second pregnancy, Jayne does not have any children as her previous pregnancy miscarried following an amniocentesis procedure, the results of which were found to be negative. Jayne had volunteered to undergo this procedure due to the increased risk of Down syndrome due to her age. A routine ultrasound examination has provided cause for concern, and Jayne has been advised to undergo another amniocentesis. Although Jayne is unwilling to undergo a further test of this type, she is concerned to know the condition of her baby as she is unsure as to whether she could cope with a child with special needs. Due to her distress, Jayne is offered the use of a pioneering technique whereby a US expert in the behavioral diagnosis of fetal abnormality could remotely scan her baby. In this way, Jayne could be provided with a test that would present no physical risk to herself or her baby.

12. Diagnostic modelling:
A cardiac unit has introduced the use of a new diagnostic package which permits the prediction of myocardial infarction using patient-reportable clinical history factors following heart surgery. It is proposed that the individual results shown by the package would affect lifestyle advice such as diet and exercise as well as prescribed medication and outpatient care.

13. Wireless technology:
Ann Young is a district nurse who uses a palmtop with wireless access to the Internet and PCT intranet. Ann regularly uses her palmtop in order to ask advice of her colleagues or to obtain test results, and she now views her palmtop as an invaluable resource. At the next practice meeting, Ann intends to present the benefits of using a palmtop to her colleagues.

14. Use of email distribution lists and discussion forums:
Rachel, a recently qualified registered general nurse (RGN), has been feeling overwhelmed by her new job and increasingly isolated since leaving her course. However, on receiving her first email at her new work address, Rachel finds that she has been placed on a distribution list for newly qualified nurses in all fields that provides contacts and professional information that would be of interest to her. Through this list, Rachel receives an invitation to join a Web-based discussion forum specifically for RGNs that would permit the sharing of experiences and nursing-related information.

15. Access to health records over the Internet:
Mohamed, 32, is completing a medical form for his new job. As his job involves traveling throughout Asia and the Far East, one of the questions on the form asks him for the dates that he has been given various vaccinations in the past. Mohamed did not keep a record of his vaccination dates and so consults his health records from his work computer in order to determine the correct dates.

16. Telematic assistance:

Sally, a nurse practitioner in charge of an asthma clinic, has been asked to trial a telematic assistance package based on ASISTASTM, which provides the nonspecialist physician with support in the diagnosis and treatment of asthma. Sally is required to evaluate the system with respect to its effectiveness and its use within the practice.

17. Knowledge source on the Internet:
An interdisciplinary committee of heath professionals has been formed to help set up an electronic data bank of materials for patients and lay people concerning AIDS, HIV, and other sexually transmitted diseases in an inner-city area. It is proposed that the data bank should include generic information about each condition and provide a knowledge resource for individuals in the local area. The basic structure has been developed for a national data bank, and it is now the job of the committee to discuss the relevant issues to the tailoring of the structure to a local context and to implement use of the data bank at a local level.

18. Data mining:
Local health authorities are seeking guidance on policy concerning data mining of electronic records by the police. The police are requesting access to protected health information in order to aid them in solving cases and making police checks.

19. Use of a probabilistic belief network:
Harry, a consultant cardiologist, has been asked to evaluate the use of a probabilistic belief network within his department entitled The Heart Disease Programme. This network provides an “intellectual sounding board” for the differential diagnosis of heart conditions and the effects of therapy, and it has been trialed within the department for six months.

20. Diagnosis/teleconferencing:
Jack is nine years old and has been officially classified as having special education needs since he entered school at the age of 4. Despite his mother’s insistence that a diagnosis should be made, none of the health professionals that have come into contact with Jack appear to be able to decide on an appropriate diagnosis or, moreover, if diagnosis itself is appropriate. Through her reading, Jack’s mother discovers an email address of a pediatrician who provides diagnoses over the Internet through email and teleconferencing. As Jack’s mother can contact the pediatrician directly, a referral by Jack’s GP is not required. The pediatrician is provided with electronic information regarding Jack’s characteristics and “symptoms” and speaks to Jack and his mother via teleconferencing. Following a number of teleconsultations, Jack is diagnosed with attention deficit hyperactivity disorder (ADHD) and prescribed the drug ritalin by his GP.

21. Web-based health information resource for older people and their care givers:
June, 83, and her care giver, Margaret, have recently been informed of a new website entitled Homecare that has been specifically developed as a resource for older people and their care givers. Homecare contains information regarding local and national services and initiatives, as well as current policy and guidelines on care practices.

22. Insurance database:
Asad has recently taken out a new insurance policy with an online insurance company. When completing the subform on medical history, Asad indicated that he had previously suffered from depression, which required medical treatment. Upon reading the terms and conditions of the application, Asad read that all information that he provided would be stored in an electronic database.

23. Genetic database:
Permission has recently been granted for the development and implementation of a genetic database that would record the genetic details of a large group of volunteers. An independent body is now charged with dealing with proposals for the use and restrictions on the use of the information contained in this database.

24. Email consultation:
A GP has set up a regular period each week for email consultations with her patients. Patients are advised to email their GP with any nonurgent concerns, and the GP will reply to their message during the designated time period. The times that this service is available are well advertised in the surgery, and more GPs within the practice are following suit.
